# A Systematic Review of the Safety of Blocking the IL-1 System in Human Pregnancy

**DOI:** 10.3390/jcm11010225

**Published:** 2021-12-31

**Authors:** Marie-Eve Brien, Virginie Gaudreault, Katia Hughes, Dexter J. L. Hayes, Alexander E. P. Heazell, Sylvie Girard

**Affiliations:** 1 Ste-Justine Hospital Research Center, Montreal, QC H3T 1C5, Canada; marie-eve.brien.hsj@ssss.gouv.qc.ca (M.-E.B.); virginie.gaudreault7@gmail.com (V.G.); khugh049@uottawa.ca (K.H.); 2 Maternal and Fetal Health Research Centre, Faculty of Biology, Medicine and Health, University of Manchester, Manchester Academic Health Science Centre, Manchester M13 9PL, UK; dexter.hayes@manchester.ac.uk (D.J.L.H.); alexander.heazell@manchester.ac.uk (A.E.P.H.); 3 Department of Obstetrics and Gynecology, Universite de Montreal, Montreal, QC H3T 1J4, Canada; 4 Department of Obstetrics and Gynecology, Department of Immunology, Mayo Clinic, Rochester, MN 55902, USA

**Keywords:** IL-1 blockade, anakinra, canakinumab, pregnancy, human, inflammation

## Abstract

Blockade of the interleukin-1 (IL-1) pathway has been used therapeutically in several inflammatory diseases including arthritis and cryopyrin-associated periodic syndrome (CAPS). These conditions frequently affect women of childbearing age and continued usage of IL-1 specific treatments throughout pregnancy has been reported. IL-1 is involved in pregnancy complications and its blockade could have therapeutic potential. We systematically reviewed all reported cases of IL-1 blockade in human pregnancy to assess safety and perinatal outcomes. We searched several databases to find reports of specific blockade of the IL-1 pathway at any stage of pregnancy, excluding broad spectrum or non-specific anti-inflammatory intervention. Our literature search generated 2439 references of which 22 studies included, following extensive review. From these, 88 different pregnancies were assessed. Most (64.8%) resulted in healthy term deliveries without any obstetrical/neonatal complications. Including pregnancy exposed to Anakinra or Canakinumab, 12 (15.0%) resulted in preterm birth and one stillbirth occurred. Regarding neonatal complications, 2 cases of renal agenesis (2.5%) were observed, and 6 infants were diagnosed with CAPS (7.5%). In conclusion, this systematic review describes that IL-1 blockade during pregnancy is not associated with increased adverse perinatal outcomes, considering that treated women all presented an inflammatory disease associated with elevated risk of pregnancy complications.

## 1. Introduction

Pregnancy complications are often associated with inflammation at the maternal-fetal interface. During pregnancy complication, such as preeclampsia (PE), preterm birth (PTB) and fetal growth restriction (FGR), inflammation can be found in the maternal circulation as well as in the placenta. Uncontrolled inflammation can negatively affect placental function [1,2,3,4,5,6]. Any alteration in placental function is associated with neonatal complications and altered child development particularly neurodevelopmental delay [7,8,9,10]. Therapeutically targeting inflammation in pregnancy has been challenging since inflammatory processes are also involved in physiological pregnancies, especially at the time of implantation and parturition [11,12,13,14,15,16] and a proinflammatory profile can be observed toward the end of uncomplicated pregnancy [16,17,18]. Therefore, there is a need to differentiate between physiological and pathological inflammation in order to develop and apply novel anti-inflammatory strategies in the clinical setting.

Inflammation has been observed at all stages of pregnancy [19,20,21,22,23,24,25,26]. Inflammation can occur in response to bacterial or viral infections (collectively referred to as pathogens-associated molecular patterns—PAMPs), as well as in response to sterile or endogenous mediators, termed damage-associated molecular pattern—DAMPs or alarmins, the latter increasingly associated with pathological pregnancies [27,28,29,30,31,32]. In order to mitigate the effects of dysregulated inflammation during pregnancy, multiple broad-spectrum anti-inflammatory therapies have been used and developed with interesting results [33,34]. Amongst these therapies, corticosteroids and nonsteroidal anti-inflammatory drugs (NSAIDs) are the most prevalent. However, studies have investigated the effect of corticosteroids during pregnancy and found an association between corticosteroid use and pregnancy/neonatal complications [35,36,37,38]. In a systematic review, Bandoli G. et al. found little to no association between corticosteroids and adverse pregnancy outcomes [35] whilst other groups reported concerns such as higher rate of cerebral palsy among children who had been exposed to repeated doses of corticosteroids or impaired growth of the lung parenchyma in cases of treatment without premature birth [36,37,38]. As for NSAIDs, acting through the inhibition of cyclooxygenase enzymes (COX-1 and COX-2), a study by Bérard A. et al., showed elevated risk of prematurity associated with the use of COX-2 inhibitors [39]. However, NSAIDs use during the first trimester of pregnancy was not associated with congenital malformations [40].

In case of pregnancies complicated with infections, antibiotics are often used. However, several studies have shown the detrimental effects of antibiotics on the development of the newborn. In the ORACLE series of clinical studies, the use of some antibiotics in pregnancy has been associated with elevated incidence of neurodevelopmental disorders [41,42]. Others have found increased risk of spontaneous miscarriage [43], without any association with congenital malformation [44]. All these drugs have benefits, but also major risk associated with their use. Since these are not specific and affect several inflammatory pathways at once, it is possible that pathways important in physiological pregnancy are impacted and responsible for the side effects observed. Targeting specific inflammatory mediators/pathways involved in pathological pregnancies could provide an efficient mean to mitigate the negative impact of inflammation, subsequently protecting the placenta and developing fetus, whilst having less deleterious effects.

The interleukin-1 (IL-1) system has been consistently associated with pregnancy complications such as preterm birth, including chorioamnionitis, FGR and PE [1,5,6,32,45,46,47,48,49] as well as high-risk pregnancies with reduced fetal movements [50]. The IL-1 system has been targeted in several animal models of pregnancy complications and blockade of this pathway appears to reduce the incidence of complications and protect the placenta as well as both fetal/neonatal development [4,51,52,53,54,55,56,57,58] and as reviewed previously by us, with emphasis on means of blocking the IL-1 pathway and their mechanisms of action with schematic representation [59]. Aside from studies in animal models, Il-1 blockers have been used for many years to help mitigate/resolve inflammatory conditions in humans [60,61,62,63]. The IL-1 receptor antagonist, IL-1Ra, is the most commonly used IL-1 system antagonist and is known under the generic name Anakinra (brand name Kineret). Anakinra has been approved for clinical use for over 20 years and has been commonly used for several chronic inflammatory conditions (such as arthritis and lupus) and in the pediatric population [60,64,65,66,67,68]. Canakinumab, brand name Ilaris, is a monoclonal antibody targeting IL-1β which has been approved for inflammatory condition such as cryopyrin-associated periodic syndromes (CAPS), since 2009 [69,70]. These inflammatory conditions commonly affect women of reproductive age and continued usage of Anakinra and/or Canakinumab during pregnancy has been reported [71,72,73]. Despite their wide range of beneficial effects, these drugs are not yet approved for use in pregnant women and are used solely when the benefit of continuing the treatment during the pregnancy outweigh the risk.

In light of the important need for targeted anti-inflammatory therapies during pregnancy, the evidence that the IL-1 system is central to both PAMPs and DAMPs-induced inflammation at the maternal-fetal interface, our objective was to perform a systematic review of all reports of any specific blockers of the IL-1 system being used during human pregnancy, to assess their potential impact on pregnancy outcome and neonatal health.

## 2. Materials and Methods

The systematic review is reported in accordance with Preferred Reporting Items for Systematic Reviews and Meta-Analyses (PRISMA) guidelines [74]. The review protocol was registered with the International Prospective Register of Systematic Reviews (PROSPERO) on 6 July 2020 (CRD42020197186).

### 2.1. Information Sources, Search Strategy and Eligibility Criteria

Literature searches were conducted in PubMed, EMBASE, MEDLINE, Cochrane Database of Systematic Reviews and Google Scholar. The search was not limited by dates but was limited to titles, abstracts and manuscripts written in English and French (for practical reasons). Reviews were excluded to ensure inclusion of original research only. Abstract from conferences were included as well, unless the same data was published and therefore only the final research article was included to avoid duplication of the same cases. Reference lists of included studies were checked for any other relevant papers. Manuscripts were identified with the search terms ’pregnancy’ and ’IL-1 blockage’ or ’IL-1 blockade’ or ‘IL-1 receptor agonist’ or ‘IL-1ra’ or ‘Anakinra’ or ‘Kineret’ or ‘Rilonacept’ or ‘Canakinumab’ or ‘Rytvela’. All searches were completed by 9 July 2021. An example search is included in Data S1.

We included cohort studies (prospective and retrospective), case series and case reports which reported the used of IL-1 blockage during pregnancy. We included all studies involving pregnant individuals who received IL-1 blockage at any stage during their pregnancy.

Our main objective was to document pregnancy outcomes related to treatment (i.e., IL-1 blockade) with IL-1 antagonists during pregnancy. The medical indication for the treatment, chronic inflammatory pathologies diagnosed prior to pregnancy in most cases, was also considered. Data regarding the rates of pregnancy complications (including: congenital anomalies, hypertensive disorders of pregnancy, preterm birth—delivery before 37 weeks of gestation, FGR, neonatal and maternal death) were extracted. We compared all these outcomes to the reported incidence in the general population and population of women with inflammatory pathologies.

### 2.2. Data Extraction

Duplicates were removed, and all citations were screened for relevance using the full abstract and indexing terms. Two out of three reviewers (MEB, VG or KH) had to agree that a study for it to be included, according to the pre-specified inclusion and exclusion criteria. When available, full-length manuscripts were obtained. Two reviewers (MEB and VG) made final inclusions decisions independently and a third reviewer (SG) was consulted to resolve any conflict when necessary.

### 2.3. Assessment of Risk of Bias and Methodological Quality

Included cohort studies were assessed using the Risk of Bias in Non-randomised Studies—of Interventions (ROBINS-I) with 7 domains, since all the studies included in this systematic review were observational. This method categorises each study by low, moderate, serious, critical risk of bias or no information [75]. If a study’ risk of bias was categorised as serious or critical, the effect of removing this study was tested and the relevant outcome reported. Individual case reports were assessed using a specific tool to assess the methodological quality of case reports [76]. This assesses 8 characteristics in 4 domains of selection, ascertainment, causality and reporting.

### 2.4. Data Synthesis

Studies with continuous data (i.e., birthweight and gestational age) were taken to obtain overall means and standard deviations. It was intended to investigate effect of exposure to IL-1 blockade at different times of pregnancy, but data could not be stratified by trimester of exposure since too many data were missing.

## 3. Results

**Figure 1:**
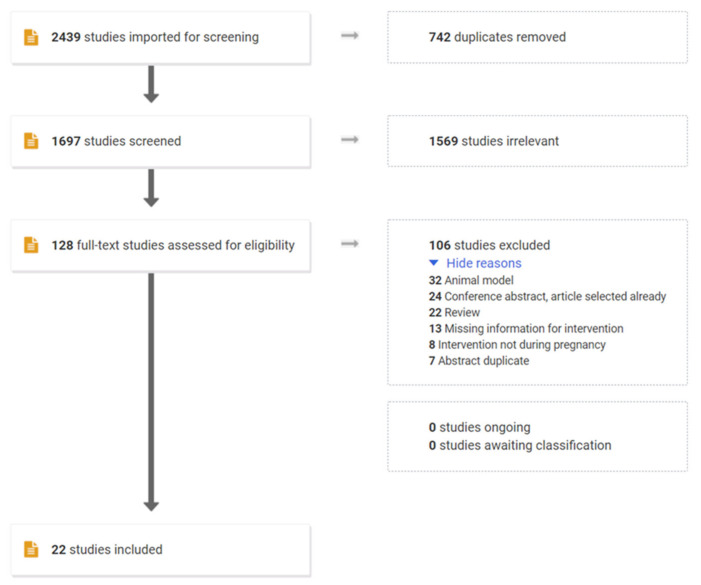
PRISMA flow chart of the systemic review of studies investigating the effect of IL-1 blockage during pregnancy.

The search strategy (Figure 1) identified 2439 articles. After removing duplicates (n = 742), 1697 papers were screened based on their title and abstracts. 1569 papers were excluded based of irrelevant to the question, exposure not during pregnancy and IL-1 blockade effects were not reported. On this basis, resulting in 128 papers for which full text was evaluated. 106 studies were excluded as they were reviews, conference abstract with original article already included, missing information or reports of animal studies, meaning 22 papers were included in the final synthesis. The 22 included studies were 9 case reports, 13 cohort studies (6 retrospective and 7 prospective).

### 3.1. Risk of Bias/Methodological Quality of Included Studies

The majority of studies included in this systematic review had a low risk of bias in the assessed domains as evaluated with the ROBINS-I tool. The majority of the case reports included adequate case ascertainment and follow-up, but there was limited data about the causal relationship between exposure to Anakinra and Canakinumab and adverse reactions (Table S1). It is important to note that almost half of the studies included in this systematic review were published conference abstracts and therefore provided limited data which could impact the results presented. Furthermore, five pregnancies were exposed to both Anakinra and Canakinumab which could affect the classification of the intervention and their outcomes.

### 3.2. Study Characteristics

Characteristics of each study and summary of findings are presented in Table 1. Within the 22 studies included, 88 individual pregnancies were reported. 75 pregnancies (85.2%) received Anakinra and 13 (14.8%) Canakinumab. Of these 88 pregnancies, 5.7% were exposed to both agents over the course of their pregnancy. The indications for these treatments were mostly cryopyrin-associated periodic syndrome—CAPS (34.1%), including familial cold autoinflammatory syndrome—FCAS, neonatal-onset multisystem inflammatory disorder—NOMID and Muckle-Wells syndrome—MWS; familial Mediterranean fever—FMF (33.0%); and adult-onset Still’s disease—AOSD or systemic juvenile idiopathic arthritis—SJIA (20.4%). The remaining cases (11 women/12.5%) received treatment for the “TNF receptor associated periodic syndrome”—TRAPS (3.4%), haemophagocytic lymphohistiocytosis—HLH (2.3%) or other pathologies such as idiopathic pericarditis, Cogan syndrome or chronic inflammatory rheumatic disease (6.8%).

Of the 88 pregnancies, 4 women (4.5%) were still pregnant at the time of publication without any follow up available for their pregnancies. Of these women, three were within their first trimester and one in the second trimester, all without any complication reported to date. In the rest of pregnancies, three (3.4%) resulted in miscarriage during the first trimester (two exposed to Anakinra and one to Canakinumab). Two of these spontaneous miscarriages occurred in the same patient, the first whilst on Canakinumab and the second with Anakinra since she presented with refractory Cogan syndrome. Unfortunately, the patient only had a partial clinical and biochemical response of her underlying diseases despite dose escalation of both treatment regimens. Finally, one patient on Anakinra terminated her pregnancy electively. For the rest of the analysis, these patients were excluded due to the lack of information.

### 3.3. Duration of Exposure to Drugs during Pregnancy

**Figure 2:**
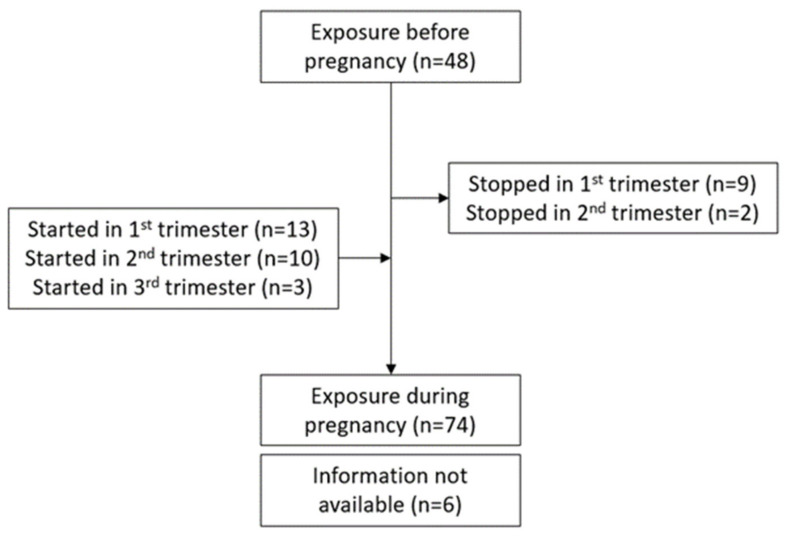
Flow chart of IL-1 blockade exposure during pregnancy (for the 11 pregnancies that stopped treatment during their 1st or 2nd trimester, treatment was initiated before conception and therefore they were included in the n = 48).

In 48 cases (60.0%) of the remaining 80 pregnancies, the women were already taking the medication prior to getting pregnant; in 6 cases, this was unknown. In 50 cases (62.5%), the drug therapy was continued throughout pregnancy (when it was started either prior to or during the first trimester until birth). In nine cases, treatment was stopped after the first trimester and, in 2 cases, after the second trimester, due to the lack of data on safety of these drugs in pregnancy. In 13 cases, treatment was started either during the second half of pregnancy (10 patients) or during the third trimester (3 patients). Of these 13 cases, 4 were due to a lack of improvement with their previous treatment (i.e., colchicine or prednisone), 2 women were diagnosed with AOSD or HLH while pregnant and no information was given for the remaining seven women. Details of the treatment duration is shown in a flow chart (Figure 2). Information concerning each pregnancy separately are shown in Table 2.

### 3.4. Anakinra Use during Pregnancy and Maternal/Fetal Outcome

Women treated with Anakinra received doses ranging from 50 to 200 mg/daily, but the majority (59.4%) received 100 mg/day. In 20 cases (29.0%), this information was unavailable.

Of the 69 pregnancies exposed to Anakinra, 63.8% had term births, 17.4% were preterm (mean gestational age: 34.1 weeks (range: 28–36.9)) and for the rest (18.8%) this information was not given. Overall, the mean gestational age at delivery in the Anakinra exposed group was 37.9 (28.0–41.1) weeks. Within Anakinra-exposed pregnancies, 63.8% had no adverse obstetric outcome and 26.1% had complications with the most predominant being preterm birth (12/18) while the rest presented one or more of the following complication; vaginal bleeding, hypertension and/or oligohydramnios. One case ended in stillbirth, and the women received Anakinra for familial Mediterranean fever from 34 weeks of gestation to the time of stillbirth (37 weeks). No additional information was available about this event. It is also important to note that one twin dichorionic-diamniotic pregnancy occurred with the demise of one fetus due to bilateral renal agenesis at 30 weeks’ gestation. However, the surviving twin had no abnormality and was born at 38.7 weeks. This pregnancy was treated from the first trimester with Anakinra for neonatal-onset multisystem inflammatory disorder. The obstetric data were not available in 10.1% of the cases.

As for the neonates, 86.4% were healthy whereas 13.6% presented some mild complications. Of these, five babies were diagnosed with CAPS whereas three presented with other problems such as hypotrophic, respiratory distress syndrome, renal agenesis, ectopic neurohypophysis, right hydrocele and/or heart murmur. Rates of breastfeeding were available for 42 pregnancies with half of them being breastfeed; however, it was not clear if the treatment was maintained during this time.

### 3.5. Canakinumab Use during Pregnancy and Maternal/Fetal Outcome

The women using Canakinumab received doses starting from a single 120 mg dose to 300 mg/8 weeks but 54.5% received 150 mg/8 weeks. Of the 11 pregnancies exposed to Canakinumab, 90.9% delivered at term and the mean gestational age in this population was 38.8 (37.0–40.0) weeks whilst for the remaining one case the information was unavailable. In this group, 90.0% had no adverse obstetrical outcome with only one woman developing gestational diabetes and one without any information.

All the babies exposed to Canakinumab were healthy with one presenting the same NLPR3 mutation as the mother. Rates of breastfeeding were available for 5 cases and 80.0% were breastfeed but again, no data on treatment during this period.

## 4. Discussion

This systematic review aimed to review the effects of IL-1 antagonists used during pregnancy in humans. We found 22 studies including 12 original articles and 10 conference abstracts published which were reporting at least one pregnancy exposed to IL-1 blockade. Of these 22 studies, data extraction was performed, and 88 different pregnancies were included in this systematic review. Furthermore, some pregnancies were reported more than once and therefore the extraction were combined to obtained complete information whilst avoiding duplicates.

Of the 88 pregnancies included, 85.2% of women received Anakinra whereas 14.8% received Canakinumab. This disparity could be due to the fact that Anakinra has been approved for therapeutic use for over 20 years as opposed to Canakinumab [65]. Furthermore, Canakinumab has a higher cost and is less widely used [77]. In a recent review by Soh and Moretto, the authors summarize the European League Against Rheumatism—EULAR and British Society on Rheumatology—BSR guidelines for biologic therapies used during pregnancy [61]. In the EULAR guidelines, Anakinra is tolerated in early pregnancy and can be continued during pregnancy if there are no other options. On the other hand, the BSR guidelines reports insufficient data to recommend the use of Anakinra during pregnancy, but stipulate that “unintentional use during first trimester is unlikely to cause harm”. Furthermore, these guidelines states that it is “not recommended to continue the treatment during gestation”. The data for Canakinumab are even more sparse. One case report measured its transplacental transfer and found a cord blood to maternal blood ratio of 2.11 [78] which needs to be further studied.

Autoimmune diseases often have negative impact on fertility and pregnancy outcomes [76,77]. Two factors can be considered to affect the course of pregnancy, the disease or the treatment for this disease. In the exposed pregnancies included in this review, the indications for treatment were CAPS (34.1%), Familial Mediterranean Fever (FMF-33%), and AOSD/SJIA (20.4%). There is only one report, to our knowledge, of CAPS during pregnancy treated with medication other than those targeting the IL-1 system [78]. This study reported a rate of miscarriage of 30% as compared to 10% for CAPS-patients treated with Anakinra [78]. Only two studies reported pregnancy with those pathologies all exposed to Anakinra or Canakinumab, therefore it is difficult to distinguish the treatment effect to that from the inflammatory pathology itself [75,78]. There is only one report of untreated pregnancies with different autoimmune disease that the one that are reported in this study making the evaluation of the pathologies themselves difficult. In this study, the authors compared pregnancy with or without all kind of DMARDs and healthy pregnancy. However, this study did not discriminate for different treatments and only three pregnancies were exposed to Anakinra [79]. Autoimmune diseases often have negative impact on fertility and pregnancy outcomes [79,80]. Two factors can be considered to affect the course of pregnancy, the disease or the treatment for this disease. In the exposed pregnancies included in this review, the indications for treatment were CAPS (34.1%), Familial Mediterranean Fever (FMF-33%), and AOSD/SJIA (20.4%). There is only one report, to our knowledge, of CAPS during pregnancy treated with medication other than those targeting the IL-1 system [81]. This study reported a rate of miscarriage of 30% as compared to 10% for CAPS-patients treated with Anakinra [81]. Only two studies reported pregnancy with those pathologies all exposed to Anakinra or Canakinumab, therefore it is difficult to distinguish the treatment effect to that from the inflammatory pathology itself [78,81]. There is only one report of untreated pregnancies with different autoimmune disease that the one that are reported in this study making the evaluation of the pathologies themselves difficult. In this study, the authors compared pregnancy with or without all kind of DMARDs and healthy pregnancy. However, this study did not discriminate for different treatments and only three pregnancies were exposed to Anakinra [82].

For patients with FMF, increased rates of miscarriage, premature rupture of membranes and low birth weight were observed compared to pregnant women without the disease [80]. In this study, 80% of women received Colchicine and none received anti-inflammatory treatment [80]. Furthermore, one retrospective study by Ben-Chetrit et al., reported an elevated rate of spontaneous abortion in untreated FMF as opposed to those treated with Colchicine [83]. Unfortunately, Colchicine resistance is often observed in FMF patients and Anakinra is increasingly used to prevent flare-ups of the disease [73]. As for AOSD, 5 case reports and 17 pregnancies were reviewed by [84]. In this cohort, most women were exposed to corticosteroid with several reported adverse outcomes such as spontaneous miscarriage observed (9.1%), premature delivery (18.2%) and FGR (9.1%). In the current systematic review, 3/14 pregnancies complicated with AOSD ended in premature delivery which is comparable to that reported by Mok et al. In a study by Garcia-Fernandez et al., on women with SJIA, 20% had preterm delivery [85], which is in contrast to the current work in which we observed no preterm delivery in SJIA with anti-IL-1 treatment. This difference could be explained by the treatment since most women in this systematic review received Anakinra as opposed to corticosteroid or other DMARDs.

We reported three miscarriages out of 88 pregnancies. All these losses occurred during the first trimester and two were exposed to Anakinra whereas one exposed to Canakinumab. This is in accordance with the literature that most miscarriages will occur during the first trimester; however, it is very difficult to measure the rate of miscarriages in the general population. Furthermore, in this cohort, two out of the three miscarriage occurs in the same women who had Cogan syndrome, a rare and severe autoimmune disease. In the literature, only eight cases of successful pregnancy with this disease have been reported [86,87,88,89,90,91]. Thus, it cannot be concluded that the therapy caused pregnancy loss in these women.

In the current work, the rate of preterm birth was 17.6% (all conditions combined) as opposed to a baseline of 11.1% [92]. However, the reported rate of preterm birth in a population with inflammatory disease is known to be higher; namely 13.6% in FMF [80], 18.1% in AOSD [84] and 20% in SJIA [85]. Although the rate of preterm birth are similar overall, it is important to keep in mind that there is no report of untreated pregnancies with those inflammatory pathologies. Only one study reported a preterm birth rate of 9% in pregnancy with inflammatory pathologies without DMARDs treatment. The maternal condition in this study were Sjögren syndrome, undifferentiated connective tissue disease (UTCD), systemic lupus erythematosus (SLE), antiphospholipid syndrome (APS) and others [82].

Our review reports neonatal complications in 13.6% of pregnancies exposed to Anakinra and 10.0% for those exposed to Canakinumab, totaling 13.2% who had complications overall. Of the 10 babies who had complications, six were diagnosed with CAPS whereas three had minor developmental delays or other problems and one died at 37 weeks (stillbirth). One baby of a FMF mother was hypotrophic, had respiratory distress syndrome and hyperbillirubilemia at birth; however, this baby was delivered prematurely at 33 + 5 weeks and was healthy at 12 months of age [93]. Baby born preterm has more neonatal complication then their counterpart born at 37 week and onwards [94,95]. Another baby born to a mother with SJIA had right hydrocele and heart murmur at birth but these complications could be due to maternal exacerbation of symptom such as oligohydramnios and hypertension. At the follow up, this baby had no major long-term complications nor malformations [96]. Finally, one baby born to a mother with active refractory AOSD had renal agenesis and ectopic neurohypophysis [97]. This is the second case of renal agenesis in Anakinra-exposed patient. The first case was in a mother diagnose with NOMID and it was a twin dichorionic-diamniotic pregnancy with fetal demise of one fetus with bilateral renal agenesis at 30 weeks. The surviving twin had no congenital abnormality and was born at 38.7 weeks. This case of congenital malformation could potentially be explained by the increased risk factor of renal tract abnormalities in twin birth as mentioned by the authors [97,98]. Furthermore, a study by Wiesel et al. reported that renal agenesis occurs in 58 of 709,030 live birth, significantly lower than 2 cases out of 88 pregnancies in this systematic review. One group has made a hypothesis that a link between uncontrolled maternal disease and renal abnormalities can occurs [96] but the potential link between renal malformation and IL-1 pathway should be the focus of future studies.

It is interesting to note that three studies, not included in the current systematic review, have evaluated 10 men who received IL-1 blockade prior to conception, resulting in 13 pregnancies. In those studies, six men had CAPS, two AOSD, one SJIA and one FMF. Seven received Anakinra (100 mg/day) and three were treated with Canakinumab (150 mg/8 weeks) at the time of conception. No adverse effect on the child wellbeing were reported after paternal exposure to IL-1 blockage [97,99,100].

This is the first systematic review to examine the effects of IL-1 blockade during pregnancy and we provide a summary of all pregnancies exposed to Anakinra or Canakinumab. Our study also highlighted the current lack of data and identified research gaps to be addressed, particularly the difference between the effects on pregnancy of the inflammatory pathology being treated as compared to the treatment itself. Our study was limited by the fact that abstracts from conferences were also included, in order to cover all exposed pregnancies, but some information were missing in relation to doses and outcomes in these abstracts.

## 5. Conclusions

In conclusion, this review summarizes all the pregnancy exposed to Il-1 blockage and no major obstetrical and neonatal complication was reported. Il-1 blockage during pregnancy could be safe and beneficial in cases of pregnancy with inflammatory conditions.

## Figures and Tables

**Table 1:** Characteristics and summary from studies included.

Study	Study Design	Year of Publication	Population	Number of Pregnancies Included in This Study	Indication for Treatment	Treatment; Doses	Outcome	Notes
1	Case report	2009	1	1	AOSD	Anakinra; 100 mg/day	Healthy term baby	
2	Case report	2011	2	2	AOSD	Anakinra; 100 mg/day	Two healthy babies, one PTB 36 weeks	
3	Retrospective cohort study	2013	51	1	SOJIA	Anakinra; NA	Term baby	Big cohort of SOJIA patients but only one pregnant
4	Prospective cohort study	2014	9	9	FCAS (6) NOMID (1) MWS/NOMID (1)	Anakinra; mostly 100 mg/day but also 239–300 mg/day	All term babies, three with FCAS, one with MWS and one twin pregnancy resulted in one death at 30 weeks	
5	Prospective cohort study	2015	4	0	FMF	Anakinra; 100 mg/day	All healthy babies, one PTB 36 weeks	Data in another study already included
6	Prospective cohort study	2015	6	3	FMF	Anakinra; 100 mg/day or NA	All healthy babies, one PTB 36 weeks	Data in another study already included
7	Prospective cohort study	2015	79	1	Chronic inflammatory rheumatic disease	Anakinra; NA	Voluntary pregnancy termination	Big cohort of biological drug during pregnancy, only one took anakinra
8	Case report	2017	1	1	FMF	Anakinra; 100 mg/day	Healthy term baby	
9	Prospective cohort study	2018	5	5	AOSD (3) SOJIA (2)	Anakinra; 100 mg/day	All healthy term babies but one with right hydrocele, heart murmur and resolved low birthweight	
10	Retrospective cohort study	2018	4	4	FMF	Anakinra; 100 mg/day–2 days	All healthy babies but one PTB at 33 weeks with hypotrophic, respiratory distress syndrome, hyperbilirubinemia and poor drinking	
11	Prospective cohort study	2019	13	12	FMF	Anakinra; 100 mg/day or NA	One miscarriage, two PTB, one stillbirth but overall healthy babies	Two pregnancies still ongoing, no obstetrical information and two pregnancies with data in another study already included
12	Case report	2019	1	1	FMF	Anakinra; 100 mg/day	Term healthy baby	Cohort of four patients with FMF, only one pregnant
13	Prospective cohort study	2019	54	1	FMF	Anakinra; 100 mg/day	Obstetrical and neonatal information NA	Cohort of patient with FMF, only one pregnant
14	Case report	2019	1	1	HLH	Anakinra; 200 mg/twice daily	Healthy but had anaemia and marrow suppression	
15	Retrospective cohort study	2020	16	3	AOSD	Anakinra; NA	All healthy babies but one had PTB at 28 weeks	Cohort of child exposed to DMARDs, only 3 exposed to anakinra during pregnancy
16	Case report	2020	1	1	HLH	Anakinra; NA	PTB at 31 weeks and IUGR but overall healthy	
17	Case report	2017	1	1	MWS	Canakinumab; 150 mg/ 4–8 weeks	Healthy term baby	
18	Case report	2018	1	1	SOJIA	Canakinumab; NA	Healthy term baby	
19	Retrospective cohort study	2020	23	1	FMF	Canakinumab; 150 mg/ 6–8 weeks	One healthy term pregnancy and one without information	Cohort of patient with FMF, only 2 pregnant
20	Retrospective cohort study	2013	7	7	AOSD (1) CAPS (3) TRAPS (1) FMF (1) Idiopathic pericarditis (1)	Anakinra; NA (6), Canakinumab; NA (1)	All healthy babies, one PTB 36 weeks and one with unilateral reduced hearing at 6 weeks	Two pregnancies still ongoing, no obstetrical information
21	Case report	2015	1	1	MWS	Canakinumab; NA and Anakinra; NA	Healthy but with CAPS	
22	Retrospective cohort study	2017	43	31	AOSD (4) CAPS (16) Cogan syndrome (2) FMF (5) Idiopathic pericarditis (1) TRAPS (2) Un-SAID (1)	Anakinra; mostly 100 mg/day but also 50–300 mg/day Canakinumab; 150 mg/ 4–8 weeks	Two miscarriage (same women), two PTB, all healthy babies but one with left renal agenesis and ectopic neurohypophysis with hormone deficiency	43 pregnancies exposed to IL-1 inhibitor but 11 were male exposure

HLH: hemophagocytic lymphohistiocytosis, FMF: familial mediterranean fever, AOSD: adult-onset Still’s disease, FCAS: familial cold autoinflammatory Syndrome, NOMID: neonatal-onset multisystem inflammatory disease, MWS: Muckle-Wells syndrome, sJIA: systemic juvenile idiopathic arthritis, CAPS: cryopyrin-associated autoinflammatory syndromes, NA: not available.

**Table 2:** Pregnancies details with maternal characteristic and neonatal outcomes.

Pregnancy ID	Pregnancy from Study	Indication for Treatment	Treatment	Doses	Exposure Time	Mode of Delivery	GA at Delivery (Weeks)	Birth Weight (g)	Obstetric Complication	Child Sex	Child Wellbeing	Breasfeeding
1	14	HLH	Anakinra	200 mg/ twice daily	22 w–B	C- section	NA	NA	None	NA	Anaemia and bone marrow suppression	NA
2	19	FMF + amyloidosis	Canakinumab	150 mg/6 weeks	PC–8 w	NA	Term	NA	None	NA	Healthy	NA
3	19	FMF	Canakinumab	150 mg/8 weeks	PC–PPT	NA	NA	NA	NA	NA	NA	NA
4 *	7	Chronic inflammatory rheumatic disease	Anakinra	NA	NA	Vaginal	NA	NA	Voluntary abortion	NA	NA	NA
5	1	AOSD	Anakinra	100 mg/ day	PC–B	Vaginal	40.7	2700	Placental retention requiring manual abruption	F	Healthy	Yes
6	4	FCAS	Anakinra	100 mg/ day	PC–B	Vaginal	41.0	3742	None	NA	Healthy	No
7	4	FCAS	Anakinra	100 mg/ day	PC–B	Vaginal	41.0	3629	None	NA	FCAS	No
8	4	FCAS	Anakinra	100 mg/ day	PC–B	Vaginal	38.0	3402	None	NA	FCAS	Yes
9	4	FCAS	Anakinra	100 mg/ day	PC–B	Vaginal	37.0	3459	None	NA	Healthy	No
10	4	FCAS	Anakinra	100 mg/ day	PC–B	Vaginal	37.7	2977	None	NA	FCAS	No
11	4	FCAS	Anakinra	100 mg/ day	PC–B	Vaginal	39.0	3345	None	NA	Healthy	No
12	4	NOMID	Anakinra	300 mg/ day	PC–B	C- section	40.0	4139	Chronic hypertension	NA	Healthy	Yes
13	4	NOMID	Anakinra	239–300 mg/ day	PC–B	Vaginal	A: 38.7 B: 30.0	A: 2637 B: NA	A: None B: PTB	NA	A: Healthy B: Renal agenesis (death)	A: Yes B: No
14	4	MWS/NOMID	Anakinra	100 mg/ day	PC–B	C- section	Term	3515	None	NA	MWS	No
15	15	AOSD	Anakinra	NA	PC–B	C- section	28.0	1175	PTB	F	Healthy	NA
16	15	AOSD	Anakinra	NA	PC–B	Vaginal	40.0	3480	None	M	Healthy	NA
17	15	AOSD	Anakinra	NA	PC–B	Vaginal	38.0	3450	None	M	Healthy	NA
19	12	FMF	Anakinra	100 mg/ day	6 w–B	C- section	Term	3340	None	F	Healthy	Yes
20	17	MWS	Canakinumab	150 mg/ 8 weeks, then every 4–5 weeks	PC–34 w	C- section	39.0	2994	None	F	Healthy with NLRP3 mutation	NA
22	11	FMF	Anakinra	100 mg/ day	PC–29 w + 33 w–B	C- section	38.0	NA	Incision site infection in postpartum	M	Healthy	NA
24	11	FMF	Anakinra	NA	16 w–B	C- section	31.0	NA	PTB	F-F twins	Healthy	NA
25	11	FMF	Anakinra	NA	23 w–B	C- section	37.0	NA	NA	F	Healthy	NA
26	11	FMF	Anakinra	NA	32 w–B	C- section	40.0	NA	NA	F	Healthy	NA
27	11	FMF	Anakinra	NA	PC–B with 1 month interruption	C- section	38.0	NA	NA	F	Healthy	NA
28	11	FMF	Anakinra	NA	34 w–B	Vaginal	37.0	NA	Stillbirth	M		NA
29	11	FMF	Anakinra	NA	6 w–B	C- section	36.0	NA	PTB	F	Healthy	NA
30	11	FMF	Anakinra	NA	NA	NA	NA	NA	NA	NA	NA	NA
31 *	11	FMF	Anakinra	NA	5 w–8 w (ongoing)	NA	NA	NA	NA	NA	NA	NA
32 *	11	FMF	Anakinra	NA	PC–8 w (ongoing)	NA	NA	NA	NA	NA	NA	NA
33	2	AOSD	Anakinra	100 mg/ day	PC–B	Vaginal	39.0	3100	None	M	Healthy	No
34	2	AOSD	Anakinra	NA	12 w–B	C- section	36.0	2800	PTB	M	Healthy	No
35	18	sJIA	Canakinumab	NA	PC–35 w	Vaginal	39.0	NA	Forceps + minor episiotomy wound infection	M	Healthy	NA
36	21	MWS	Canakinumab and Ana kinra	NA	PC–B	NA	NA	NA	NA	M	Healthy with CAPS	Yes
37	8	FMF	Anakinra	100 mg/ day	PC–B	C- section	38.0	2700	None	NA	Healthy	Yes
38 *	20	CAPS	Anakinra	NA	PC–NA (ongoing)	NA	NA	NA	NA	NA	NA	NA
39 *	20	CAPS	Canakinumab	NA	PC–8 w (ongoing)	NA	NA	NA	NA	NA	NA	NA
40	20	CAPS	Anakinra	NA	PC–B	Vaginal	NA	NA	None	M	Healthy	No
41	20	TRAPS	Anakinra	NA	PC–B	Vaginal	NA	NA	None	M	Unilateral reduced hearing at 6 weeks	No
42	20	FMF	Anakinra	100 mg/ day	21 w–B	C- section	36.0	NA	Vaginal bleeding, PTB	M	Healthy	Yes
43	20	idiopathic pericarditis	Anakinra	NA	PC–B	Vaginal	NA	NA	None	M	Healthy	No
44	20	AOSD	Anakinra	NA	22 w–33 w	Vaginal	NA	NA	None	M	Healthy	No
50	6	FMF	Anakinra	100 mg/ day	12 w–B	Vaginal	40.0	NA	None	F	Healthy	Yes
52	6	FMF	Anakinra	NA	15 w–B	Vaginal	38.0	NA	None	M	Low thrombocyte count treated by IVIG	NA
54	3	sJIA	Anakinra	NA	P–B	NA	Term	NA	NA	NA	NA	NA
55	13	FMF	Anakinra	100 mg/ day	P–B	NA	NA	NA	None	NA	NA	NA
56	9	sJIA	Anakinra	100 mg/ day	PC–20.4 w	C- section	37.1	2419	Hypertension, oligohydramnios, breech presentation	M	Jaundice, right hydrocele and heart murmur	No
57	9	AOSD	Anakinra	100 mg/ day	20 w–38.1 w	Vaginal	40.1	2940	None	M	Jaundice	NA
58	9	AOSD	Anakinra	100 mg/ day	PC– 16.6 w + 19.4 w– 37.3 w	C- section	39.4	3632	None	M	Jaundice	Yes
59	9	AOSD	Anakinra	100 mg/ day	PC– 2 w + 9.6 w– 36.7 w	Vaginal	38.7	3519	None	M	Tongue-tied	Yes
60	9	sJIA	Anakinra	100 mg/ day	PC– 37.3 w	Vaginal	39.4	2640	Oligohydramnios	F	Healthy	No
61	10	FMF	Anakinra	100 mg/ day	P–B	C- section	40.6	4025	None	NA	Healthy	Yes
62	10	FMF	Anakinra	100 mg/ day	2e trimester–B	C- section	33.7	3320	PTB	NA	Healthy, hypotrophic, respiratory distress syndrome, hyperbilirubinemia and poor drinking	No
63	10	FMF	Anakinra	100 mg/2 days	P–B	C- section	39.3	4030	Premature bleeding	NA	Healthy	NA
64	10	FMF	Anakinra	100 mg/2 days	P–B	C- section	36.4	3320	PTB	NA	Healthy	NA
66	16	HLH	Anakinra	NA	22 w–B	C- section	31.7	NA	PTB, IUGR, abnormal umbilical artery Doppler and subsequent cardiotocography was abnormal	M	Neonatal unit briefly but healthy	NA
67	22	CAPS	Canakinumab	150 mg/ 8 weeks	PC–8 w	C- section	38.0	3540	Gestationnal diabetes	M	Healthy	No
68	22	CAPS	Canakinumab	150 mg/ 8 weeks	PC–12 w	Vaginal	40.0	4480	None	F	Healthy	Yes
69	22	CAPS	Canakinumab	150 mg/ 8 weeks	1 w–36 w	NA	40.0	3570	None	M	Healthy	NA
70	22	CAPS	Canakinumab	120 mg (single dose)	P	NA	38.0	3290	None	M	Healthy	Yes
71	22	Un-SAID	Canakinumab	300 mg/ 8 weeks	PC–B	Vaginal	39.0	NA	None	M	Healthy	NA
72	22	FMF	Canakinumab	150 mg/ 4 weeks	PC–B	C- section	37.0	3300	None	M	Healthy	Yes
73	22	FMF	Canakinumab	150 mg/ 8 weeks	PC–4 w	C- section	40.0	3300	None	F	Healthy	Yes
74 *	22	Cogan syndrome	Canakinumab	150 mg/ 4 weeks	PC–4 w	Vaginal	4.0	NA	Miscarriage	NA		NA
75	22	CAPS	Anakinra	50 mg/ day	PC–B	Vaginal	39.0	3940	None	M	Healthy	No
76	22	CAPS	Anakinra	50 mg/ day	PC–B	Vaginal	39.0	NA	None	F	Healthy	No
77	22	CAPS	Anakinra	100 mg/ day	PC–B	Vaginal	41.1	3600	None	M	Healthy	Yes
78	22	CAPS	Anakinra	100 mg/ day	PPT–B	Vaginal	40.0	4480	None	F	Healthy	Yes
79	22	CAPS	Anakinra	100 mg/ day	36 w–B	NA	40.0	3570	None	M	Healthy	NA
80	22	CAPS	Anakinra	100 mg/ day	1 w–PPT	NA	36.9	2830	PTB	M	Healthy	No
81	22	CAPS	Anakinra	100 mg/ day	PC–B	C- section	38.9	NA	C-section due to failure to progress	NA	Healthy	NA
82	22	CAPS	Anakinra	100 mg/ day	PC–6 w	C- section	40.0	NA	None	M	Healthy	NA
83	22	CAPS	Anakinra	100 mg/ day	PC–B	NA	NA	NA	None	M	Healthy	Yes
84	22	CAPS	Anakinra	100 mg/ day	NA	NA	40.1	NA	None	F	Healthy	NA
85	22	CAPS	Anakinra	100 mg/ day	NA	NA	NA	NA	None	F	Healthy	NA
86	22	CAPS	Anakinra	100 mg/ day	NA	NA	NA	NA	None	F	Healthy	NA
87	22	FMF	Anakinra	100 mg/ day	PC–B	C- section	36.1	2170	Vaginal bleeding, PTB	M	Healthy	Yes
88	22	FMF	Anakinra	100 mg/ day	12 w–B	Vaginal	40.0	3170	None	F	Healthy	Yes
89	22	FMF	Anakinra	100 mg/ day	PC–B	Vaginal	36.0	1600	PTB	F	Healthy	Yes
90	22	idiopathic pericarditis	Anakinra	100 mg/ day	PC–PPT	Vaginal	38.3	2930	None	M	Healthy	No
91	22	AOSD	Anakinra	200–300 mg/ day	PC–16 w	NA	37.0	2450	None	F	Healthy	No
92	22	AOSD	Anakinra	100 mg/ day	22 w– 33 w	NA	35.1	2020	PTB	M	Healthy	Yes
93	22	AOSD	Anakinra	100 mg/ day	9 w–B	C- section	38.1	NA	None	M	Left renal agenesis	Yes
94	22	AOSD	Anakinra	100 mg/ day	NA	Vaginal	38.0	3060	None	F	Healthy	Yes
95	22	TRAPS	Anakinra	100 mg/ day	PC–B	Vaginal	41.0	3230	None	M	Healthy	Yes
96	22	TRAPS	Anakinra	100 mg/ day	PC–B	NA	NA	NA	None	F	Healthy	NA

HLH: hemophagocytic lymphohistiocytosis, FMF: familial mediterranean fever, AOSD: adult-onset Still’s disease, FCAS: familial cold autoinflammatory Syndrome, NOMID: neonatal-onset multisystem inflammatory disease, MWS: Muckle-Wells syndrome, sJIA: systemic juvenile idiopathic arthritis, CAPS: cryopyrin-associated autoinflammatory syndromes, NA: not available, PC: prior to conception, B: birth, PPT: pregnancy positive test, P: pregnancy, GA: gestational age, PTB: preterm birth, IUGR: intra-uterine growth restriction, M: male, F: female, *: Excluded from further analysis since data missing.

## Supplementary Materials

The following are available online at https://www.mdpi.com/article/10.3390/jcm11010225/s1; Data S1: Search strategy used for this systematic review. Table S1: Search strategy used for this systematic review.

## References

[1] Aye I.L., Jansson T., Powell T.L. (2013). Interleukin-1beta inhibits insulin signaling and prevents insulin-stimulated system A amino acid transport in primary human trophoblasts. Mol. Cell. Endocrinol..

[2] Bainbridge S.A., Roberts J.M., von Versen-Hoynck F., Koch J., Edmunds L., Hubel C.A. (2009). Uric acid attenuates trophoblast invasion and integration into endothelial cell monolayers. Am. J. Physiol. Cell Physiol..

[3] Bainbridge S.A., von Versen-Höynck F., Roberts J.M. (2009). Uric acid inhibits placental system A amino acid uptake. Placenta.

[4] Lei J., Vermillion M.S., Jia B., Xie H., Xie L., McLane M.W., Sheffield J.S., Pekosz A., Brown A., Klein S.L. (2019). IL-1 receptor antagonist therapy mitigates placental dysfunction and perinatal injury following Zika virus infection. JCI Insight.

[5] Mulla M.J., Myrtolli K., Potter J., Boeras C., Kavathas P.B., Sfakianaki A.K., Tadesse S., Norwitz E.R., Guller S., Abrahams V.M. (2011). Uric acid induces trophoblast IL-1beta production via the inflammasome: Implications for the pathogenesis of preeclampsia. Am. J. Reprod. Immunol..

[6] Brien M.E., Duval C., Palacios J., Boufaied I., Hudon-Thibeault A.A., Nadeau-Vallee M., Vaillancourt C., Sibley C.P., Abrahams V.M., Jones R.L. (2017). Uric Acid Crystals Induce Placental Inflammation and Alter Trophoblast Function via an IL-1-Dependent Pathway: Implications for Fetal Growth Restriction. J. Immunol..

[7] Depino A.M. (2018). Perinatal inflammation and adult psychopathology: From preclinical models to humans. Semin. Cell Dev. Biol..

[8] Hagberg H., Gressens P., Mallard C. (2012). Inflammation during fetal and neonatal life: Implications for neurologic and neuropsychiatric disease in children and adults. Ann. Neurol..

[9] Van Vliet E.O., de Kieviet J.F., van der Voorn J.P., Been J.V., Oosterlaan J., van Elburg R.M. (2012). Placental pathology and long-term neurodevelopment of very preterm infants. Am. J. Obstet. Gynecol..

[10] Neiger R. (2017). Long-Term Effects of Pregnancy Complications on Maternal Health: A Review. J. Clin. Med..

[11] Erlebacher A. (2013). Immunology of the maternal-fetal interface. Annu. Rev. Immunol..

[12] Moffett A., Loke C. (2006). Immunology of placentation in eutherian mammals. Nat. Rev. Immunol..

[13] Mor G., Cardenas I., Abrahams V., Guller S. (2011). Inflammation and pregnancy: The role of the immune system at the implantation site. Ann. N. Y. Acad. Sci..

[14] Menon R., Richardson L.S., Lappas M. (2019). Fetal membrane architecture, aging and inflammation in pregnancy and parturition. Placenta.

[15] Romero R., Espinoza J., Gonçalves L.F., Kusanovic J.P., Friel L.A., Nien J.K. (2006). Inflammation in preterm and term labour and delivery. Semin. Fetal Neonatal Med..

[16] Brien M.E., Boufaied I., Bernard N., Forest J.C., Giguere Y., Girard S. (2020). Specific inflammatory profile in each pregnancy complication: A comparative study. Am. J. Reprod. Immunol..

[17] Salazar Garcia M.D., Mobley Y., Henson J., Davies M., Skariah A., Dambaeva S., Gilman-Sachs A., Beaman K., Lampley C., Kwak-Kim J. (2018). Early pregnancy immune biomarkers in peripheral blood may predict preeclampsia. J. Reprod. Immunol..

[18] Freeman D.J., McManus F., Brown E.A., Cherry L., Norrie J., Ramsay J.E., Clark P., Walker I.D., Sattar N., Greer I.A. (2004). Short- and long-term changes in plasma inflammatory markers associated with preeclampsia. Hypertension.

[19] Ferguson K.K., Meeker J.D., McElrath T.F., Mukherjee B., Cantonwine D.E. (2017). Repeated measures of inflammation and oxidative stress biomarkers in preeclamptic and normotensive pregnancies. Am. J. Obstet. Gynecol..

[20] Redman C.W., Staff A.C. (2015). Preeclampsia, biomarkers, syncytiotrophoblast stress, and placental capacity. Am. J. Obstet. Gynecol..

[21] Taylor B.D., Ness R.B., Klebanoff M.A., Zoh R., Bass D., Hougaard D.M., Skogstrand K., Haggerty C.L. (2016). First and second trimester immune biomarkers in preeclamptic and normotensive women. Pregnancy Hypertens..

[22] Taylor B.D., Tang G., Ness R.B., Olsen J., Hougaard D.M., Skogstrand K., Roberts J.M., Haggerty C.L. (2016). Mid-pregnancy circulating immune biomarkers in women with preeclampsia and normotensive controls. Pregnancy Hypertens..

[23] Ronzoni S., Steckle V., D’Souza R., Murphy K.E., Lye S., Shynlova O. (2018). Cytokine Changes in Maternal Peripheral Blood Correlate With Time-to-Delivery in Pregnancies Complicated by Premature Prelabor Rupture of the Membranes. Reprod. Sci..

[24] Giguere Y., Masse J., Theriault S., Bujold E., Lafond J., Rousseau F., Forest J.C. (2015). Screening for pre-eclampsia early in pregnancy: Performance of a multivariable model combining clinical characteristics and biochemical markers. BJOG.

[25] Kuc S., Wortelboer E.J., van Rijn B.B., Franx A., Visser G.H., Schielen P.C. (2011). Evaluation of 7 serum biomarkers and uterine artery Doppler ultrasound for first-trimester prediction of preeclampsia: A systematic review. Obstet. Gynecol. Surv..

[26] Yu N., Cui H., Chen X., Chang Y. (2017). First trimester maternal serum analytes and second trimester uterine artery Doppler in the prediction of preeclampsia and fetal growth restriction. Taiwan J. Obstet. Gynecol..

[27] Bianchi M.E. (2007). DAMPs, PAMPs and alarmins: All we need to know about danger. J. Leukoc. Biol..

[28] Matzinger P. (2002). The danger model: A renewed sense of self. Science.

[29] Brien M.E., Baker B., Duval C., Gaudreault V., Jones R.L., Girard S. (2019). Alarmins at the maternal-fetal interface: Involvement of inflammation in placental dysfunction and pregnancy complications (1). Can. J. Physiol. Pharmacol..

[30] Nadeau-Vallee M., Obari D., Palacios J., Brien M.E., Duval C., Chemtob S., Girard S. (2016). Sterile inflammation and pregnancy complications: A review. Reproduction.

[31] Sharps M.C., Baker B.C., Guevara T., Bischof H., Jones R.L., Greenwood S.L., Heazell A.E.P. (2020). Increased placental macrophages and a pro-inflammatory profile in placentas and maternal serum in infants with a decreased growth rate in the third trimester of pregnancy. Am. J. Reprod. Immunol..

[32] Saji F., Samejima Y., Kamiura S., Sawai K., Shimoya K., Kimura T. (2000). Cytokine production in chorioamnionitis. J. Reprod. Immunol..

[33] Russo R.C., Garcia C.C., Teixeira M.M. (2010). Anti-inflammatory drug development: Broad or specific chemokine receptor antagonists?. Curr. Opin. Drug Discov. Dev..

[34] Grainger D.J., Reckless J. (2003). Broad-spectrum chemokine inhibitors (BSCIs) and their anti-inflammatory effects in vivo. Biochem. Pharmacol..

[35] Bandoli G., Palmsten K., Forbess Smith C.J., Chambers C.D. (2017). A Review of Systemic Corticosteroid Use in Pregnancy and the Risk of Select Pregnancy and Birth Outcomes. Rheum. Dis. Clin. N. Am..

[36] Shanks A.L., Grasch J.L., Quinney S.K., Haas D.M. (2019). Controversies in antenatal corticosteroids. Semin. Fetal Neonatal Med..

[37] Wapner R.J., Sorokin Y., Mele L., Johnson F., Dudley D.J., Spong C.Y., Peaceman A.M., Leveno K.J., Malone F., Caritis S.N. (2007). Long-term outcomes after repeat doses of antenatal corticosteroids. N. Engl. J. Med..

[38] Bandyopadhyay A., Slaven J.E., Evrard C., Tiller C., Haas D.M., Tepper R.S. (2020). Antenatal corticosteriods decrease forced vital capacity in infants born fullterm. Pediatr. Pulmonol..

[39] Bérard A., Sheehy O., Girard S., Zhao J.P., Bernatsky S. (2018). Risk of preterm birth following late pregnancy exposure to NSAIDs or COX-2 inhibitors. Pain.

[40] Daniel S., Matok I., Gorodischer R., Koren G., Uziel E., Wiznitzer A., Levy A. (2012). Major malformations following exposure to nonsteroidal antiinflammatory drugs during the first trimester of pregnancy. J. Rheumatol..

[41] Kenyon S., Pike K., Jones D.R., Brocklehurst P., Marlow N., Salt A., Taylor D.J. (2008). Childhood outcomes after prescription of antibiotics to pregnant women with spontaneous preterm labour: 7-year follow-up of the ORACLE II trial. Lancet.

[42] Kenyon S., Pike K., Jones D.R., Brocklehurst P., Marlow N., Salt A., Taylor D.J. (2008). Childhood outcomes after prescription of antibiotics to pregnant women with preterm rupture of the membranes: 7-year follow-up of the ORACLE I trial. Lancet.

[43] Muanda F.T., Sheehy O., Bérard A. (2017). Use of antibiotics during pregnancy and risk of spontaneous abortion. CMAJ.

[44] Muanda F.T., Sheehy O., Bérard A. (2017). Use of antibiotics during pregnancy and the risk of major congenital malformations: A population based cohort study. Br. J. Clin. Pharmacol..

[45] Reis A.S., Barboza R., Murillo O., Barateiro A., Peixoto E.P.M., Lima F.A., Gomes V.M., Dombrowski J.G., Leal V.N.C., Araujo F. (2020). Inflammasome activation and IL-1 signaling during placental malaria induce poor pregnancy outcomes. Sci. Adv..

[46] Equils O., Kellogg C., McGregor J., Gravett M., Neal-Perry G., Gabay C. (2020). The role of the IL-1 system in pregnancy and the use of IL-1 system markers to identify women at risk for pregnancy complications. Biol. Reprod..

[47] Southcombe J.H., Redman C.W., Sargent I.L., Granne I. (2015). Interleukin-1 family cytokines and their regulatory proteins in normal pregnancy and pre-eclampsia. Clin. Exp. Immunol..

[48] Licini C., Tossetta G., Avellini C., Ciarmela P., Lorenzi T., Toti P., Gesuita R., Voltolini C., Petraglia F., Castellucci M. (2016). Analysis of cell-cell junctions in human amnion and chorionic plate affected by chorioamnionitis. Histol. Histopathol..

[49] Tossetta G., Paolinelli F., Avellini C., Salvolini E., Ciarmela P., Lorenzi T., Emanuelli M., Toti P., Giuliante R., Gesuita R. (2014). IL-1β and TGF-β weaken the placental barrier through destruction of tight junctions: An in vivo and in vitro study. Placenta.

[50] Girard S., Heazell A.E., Derricott H., Allan S.M., Sibley C.P., Abrahams V.M., Jones R.L. (2014). Circulating cytokines and alarmins associated with placental inflammation in high-risk pregnancies. Am. J. Reprod. Immunol..

[51] Girard S., Sébire H., Brochu M.E., Briota S., Sarret P., Sébire G. (2012). Postnatal administration of IL-1Ra exerts neuroprotective effects following perinatal inflammation and/or hypoxic-ischemic injuries. Brain Behav. Immun..

[52] Girard S., Tremblay L., Lepage M., Sebire G. (2010). IL-1 receptor antagonist protects against placental and neurodevelopmental defects induced by maternal inflammation. J. Immunol..

[53] Leitner K., Al Shammary M., McLane M., Johnston M.V., Elovitz M.A., Burd I. (2014). IL-1 receptor blockade prevents fetal cortical brain injury but not preterm birth in a mouse model of inflammation-induced preterm birth and perinatal brain injury. Am. J. Reprod. Immunol..

[54] Nadeau-Vallee M., Chin P.Y., Belarbi L., Brien M.E., Pundir S., Berryer M.H., Beaudry-Richard A., Madaan A., Sharkey D.J., Lupien-Meilleur A. (2017). Antenatal Suppression of IL-1 Protects against Inflammation-Induced Fetal Injury and Improves Neonatal and Developmental Outcomes in Mice. J. Immunol..

[55] Nadeau-Vallee M., Quiniou C., Palacios J., Hou X., Erfani A., Madaan A., Sanchez M., Leimert K., Boudreault A., Duhamel F. (2015). Novel Noncompetitive IL-1 Receptor-Biased Ligand Prevents Infection- and Inflammation-Induced Preterm Birth. J. Immunol..

[56] McDuffie R.S., Davies J.K., Leslie K.K., Lee S., Sherman M.P., Gibbs R.S. (2001). A randomized controlled trial of interleukin-1 receptor antagonist in a rabbit model of ascending infection in pregnancy. Infect. Dis. Obstet. Gynecol..

[57] Presicce P., Park C.W., Senthamaraikannan P., Bhattacharyya S., Jackson C., Kong F., Rueda C.M., DeFranco E., Miller L.A., Hildeman D.A. (2018). IL-1 signaling mediates intrauterine inflammation and chorio-decidua neutrophil recruitment and activation. JCI Insight.

[58] Karisnan K., Bakker A.J., Song Y., Noble P.B., Pillow J.J., Pinniger G.J. (2015). Interleukin-1 receptor antagonist protects against lipopolysaccharide induced diaphragm weakness in preterm lambs. PLoS ONE.

[59] Nadeau-Vallee M., Obari D., Quiniou C., Lubell W.D., Olson D.M., Girard S., Chemtob S. (2016). A critical role of interleukin-1 in preterm labor. Cytokine Growth Factor Rev..

[60] Prieto-Peña D., Dasgupta B. (2020). Biologic agents and small-molecule inhibitors in systemic autoimmune conditions: An update. Pol. Arch. Intern. Med..

[61] Soh M.C., Moretto M. (2020). The use of biologics for autoimmune rheumatic diseases in fertility and pregnancy. Obstet. Med..

[62] Götestam Skorpen C., Hoeltzenbein M., Tincani A., Fischer-Betz R., Elefant E., Chambers C., da Silva J., Nelson-Piercy C., Cetin I., Costedoat-Chalumeau N. (2016). The EULAR points to consider for use of antirheumatic drugs before pregnancy, and during pregnancy and lactation. Ann. Rheum. Dis..

[63] Nuki G., Bresnihan B., Bear M.B., McCabe D. (2002). Long-term safety and maintenance of clinical improvement following treatment with anakinra (recombinant human interleukin-1 receptor antagonist) in patients with rheumatoid arthritis: Extension phase of a randomized, double-blind, placebo-controlled trial. Arthritis Rheum..

[64] Buckley L.F., Viscusi M.M., Van Tassell B.W., Abbate A. (2018). Interleukin-1 blockade for the treatment of pericarditis. Eur. Heart J. Cardiovasc. Pharmacother..

[65] Kary S., Burmester G.R. (2003). Anakinra: The first interleukin-1 inhibitor in the treatment of rheumatoid arthritis. Int. J. Clin. Pract..

[66] Ramírez J., Cañete J.D. (2018). Anakinra for the treatment of rheumatoid arthritis: A safety evaluation. Expert Opin. Drug Saf..

[67] Dinarello C.A., van der Meer J.W. (2013). Treating inflammation by blocking interleukin-1 in humans. Semin. Immunol..

[68] Vastert S.J., Jamilloux Y., Quartier P., Ohlman S., Osterling Koskinen L., Kullenberg T., Franck-Larsson K., Fautrel B., de Benedetti F. (2019). Anakinra in children and adults with Still’s disease. Rheumatology.

[69] Church L.D., McDermott M.F. (2009). Canakinumab, a fully-human mAb against IL-1beta for the potential treatment of inflammatory disorders. Curr. Opin. Mol. Ther..

[70] Savic S., McDermott M.F. (2009). Inflammation: Canakinumab for the cryopyrin-associated periodic syndromes. Nat. Rev. Rheumatol..

[71] Ortona E., Pierdominici M., Maselli A., Veroni C., Aloisi F., Shoenfeld Y. (2016). Sex-based differences in autoimmune diseases. Ann. Ist. Super. Sanita.

[72] Fischer-Betz R., Specker C. (2017). Pregnancy in systemic lupus erythematosus and antiphospholipid syndrome. Best Pract. Res. Clin. Rheumatol..

[73] Ugurlu S., Ergezen B., Egeli B.H., Selvi O., Ozdogan H. (2021). Anakinra treatment in patients with familial Mediterranean fever: A single-centre experience. Rheumatology.

[74] Moher D., Liberati A., Tetzlaff J., Altman D.G. (2009). Preferred reporting items for systematic reviews and meta-analyses: The PRISMA statement. PLoS Med..

[75] Sterne J.A., Hernán M.A., Reeves B.C., Savović J., Berkman N.D., Viswanathan M., Henry D., Altman D.G., Ansari M.T., Boutron I. (2016). ROBINS-I: A tool for assessing risk of bias in non-randomised studies of interventions. BMJ.

[76] Murad M.H., Sultan S., Haffar S., Bazerbachi F. (2018). Methodological quality and synthesis of case series and case reports. BMJ Evid.-Based Med..

[77] Sfriso P., Bindoli S., Doria A., Feist E., Galozzi P. (2020). Canakinumab for the treatment of adult-onset Still’s disease. Expert Rev. Clin. Immunol..

[78] Egawa M., Imai K., Mori M., Miyasaka N., Kubota T. (2017). Placental Transfer of Canakinumab in a Patient with Muckle-Wells Syndrome. J. Clin. Immunol..

[79] Mijatovic V., Hompes P.G., Wouters M.G. (2003). Familial Mediterranean fever and its implications for fertility and pregnancy. Eur. J. Obstet. Gynecol. Reprod. Biol..

[80] Yasar O., Iskender C., Kaymak O., Taflan Yaman S., Uygur D., Danisman N. (2014). Retrospective evaluation of pregnancy outcomes in women with familial Mediterranean fever. J. Matern. Fetal Neonatal Med..

[81] Chang Z., Spong C.Y., Jesus A.A., Davis M.A., Plass N., Stone D.L., Chapelle D., Hoffmann P., Kastner D.L., Barron K. (2014). Anakinra use during pregnancy in patients with cryopyrin-associated periodic syndromes (CAPS). Arthritis Rheum..

[82] De Lorenzo R., Ramirez G.A., Punzo D., Lorioli L., Rovelli R., Canti V., Barera G., Rovere-Querini P. (2020). Neonatal outcomes of children born to mothers on biological agents during pregnancy: State of the art and perspectives. Pharmacol. Res..

[83] Ben-Chetrit E., Ben-Chetrit A., Berkun Y., Ben-Chetrit E. (2010). Pregnancy outcomes in women with familial Mediterranean fever receiving colchicine: Is amniocentesis justified?. Arthritis Care Res..

[84] Mok M.Y., Lo Y., Leung P.Y., Lau C.S. (2004). Pregnancy outcome in patients with adult onset Still’s disease. J. Rheumatol..

[85] García-Fernández A., Gerardi M.C., Crisafulli F., Filippini M., Fredi M., Gorla R., Lazzaroni M.G., Lojacono A., Nalli C., Ramazzotto F. (2021). Disease course and obstetric outcomes of pregnancies in juvenile idiopathic arthritis: Are there any differences among disease subtypes? A single-centre retrospective study of prospectively followed pregnancies in a dedicated pregnancy clinic. Clin. Rheum..

[86] Bakalianou K., Salakos N., Iavazzo C., Danilidou K., Papadias K., Kondi-Pafiti A. (2008). A rare case of uneventful pregnancy in a woman with Cogan’s syndrome. Clin. Exp. Obstet. Gynecol..

[87] Currie C., Wax J.R., Pinette M.G., Blackstone J., Cartin A. (2009). Cogan’s syndrome complicating pregnancy. J. Matern. Fetal Neonatal Med..

[88] Deliveliotou A., Moustakarias T., Argeitis J., Vaggos G., Vitoratos N., Hassiakos D. (2007). Successful full-term pregnancy in a woman with Cogan’s syndrome: A case report. Clin. Rheum..

[89] Riboni F., Cosma S., Perini P.G., Benedetto C. (2016). Successful Pregnancy in a Patient with Atypical Cogan’s Syndrome. Isr. Med. Assoc. J..

[90] Tarney C.M., Wilson K., Sewell M.F. (2014). Cogan syndrome in pregnancy. Obstet. Gynecol..

[91] Venhoff N., Thiel J., Schramm M.A., Jandova I., Voll R.E., Glaser C. (2020). Case Report: Effective and Safe Treatment with Certolizumab Pegol in Pregnant Patients With Cogan’s Syndrome: A Report of Three Pregnancies in Two Patients. Front. Immunol..

[92] Blencowe H., Cousens S., Chou D., Oestergaard M., Say L., Moller A.B., Kinney M., Lawn J. (2013). Born too soon: The global epidemiology of 15 million preterm births. Reprod. Health.

[93] Venhoff N., Voll R.E., Glaser C., Thiel J. (2018). IL-1-blockade with Anakinra during pregnancy: Retrospective analysis of efficacy and safety in female patients with familial Mediterranean fever. Z. Rheum..

[94] Romero R., Dey S.K., Fisher S.J. (2014). Preterm labor: One syndrome, many causes. Science.

[95] Saigal S., Doyle L.W. (2008). An overview of mortality and sequelae of preterm birth from infancy to adulthood. Lancet.

[96] Smith C.J.F., Chambers C.D. (2018). Five successful pregnancies with antenatal anakinra exposure. Rheumatology.

[97] Youngstein T., Hoffmann P., Gül A., Lane T., Williams R., Rowczenio D.M., Ozdogan H., Ugurlu S., Ryan J., Harty L. (2017). International multi-centre study of pregnancy outcomes with interleukin-1 inhibitors. Rheumatology.

[98] Rider R.A., Stevenson D.A., Rinsky J.E., Feldkamp M.L. (2013). Association of twinning and maternal age with major structural birth defects in Utah, 1999 to 2008. Birth Defects Res. A Clin. Mol. Teratol..

[99] Viktil K.K., Engeland A., Furu K. (2009). Use of antirheumatic drugs in mothers and fathers before and during pregnancy-a population-based cohort study. Pharmacoepidemiol. Drug Saf..

[100] Drechsel P., Stüdemann K., Niewerth M., Horneff G., Fischer-Betz R., Seipelt E., Spähtling-Mestekemper S., Aries P., Zink A., Klotsche J. (2020). Pregnancy outcomes in DMARD-exposed patients with juvenile idiopathic arthritis-results from a JIA biologic registry. Rheumatology.

