# Supplementary material for: A Systematic Review of the Safety of Blocking the IL-1 System in Human Pregnancy
Source: J Clin Med. 2021 Dec 31;11(1):225. doi: 10.3390/jcm11010225 (PMC8745599; doi:10.3390/jcm11010225)
Supplement: Supplementary file 1 [file jcm-11-00225-s001.zip › jcm-1526555-supplementary.pdf]

Data S1. Search strategy:

1. pregnancy
2. (IL-1 blockage OR IL-1 blockade OR IL-1ra OR IL-1 receptor agonist)
3. (Anakinra OR Kineret OR Rilonacept OR Canakinumab OR Rytvela)
4. 2 OR 3
5. 1 AND 4

Table S1. Evaluation of quality of case report.

Study	Selection	Ascertainment		Causality				Reporting
	Does the case(s) represent the whole experience of the investigator (centre) or is the selection method unclear to the extent that other patients with similar presentation may not have been reported?	Was the exposure adequately ascertained?	Was the outcome adequately ascertained?	Were other alternative causes that may explain the observation ruled out?	Was there a challenge/ rechallenge phenomenon?	Was there a dose–response effect?	Was follow-up long enough for outcomes to occur?	Is the case(s) described with sufficient details to allow other investigators to replicate the research or to allow practitioners make inferences related to their own practice?
Berger et al., 2009	No	Yes	Yes	N/A – no adverse effect	No	No	Yes	Yes
Fischer-Betz et al., 2011	Yes	Yes	Yes	N/A – no adverse effect	No	No	Yes	Yes
Hansmann et al., 2015	Yes	No	No	Unclear	No	No	Yes	No
Egawa et al., 2017	Yes	Yes	Yes	N/A – no adverse effect	No	No	Yes	Yes
Ilgen et al., 2017	Yes	Yes	Yes	No – infant had FGR	Yes	No	Yes	Yes
Gunn et al., 2018	Yes	No	Yes	N/A – no adverse effect	No	No	Yes	No
Ali et al., 2019	Yes	Yes	Yes	No – infant had slowing growth & bone marrow suppression	No	No	Yes	Yes
Duman et al., 2019	Yes	Yes	Yes	N/A – no adverse effect	No	No	Yes	Yes
Yip et al., 2020	Yes	Yes	Yes	No – infant had FGR	No	No	Yes	Yes

N/A = Not applicable
